# Multi‐Institutional MR‐Derived Radiomics to Predict Post‐Exenteration Disease Recurrence in Patients With T4 Rectal Cancer

**DOI:** 10.1002/cam4.70699

**Published:** 2025-02-18

**Authors:** Niall J. O'Sullivan, Fariba Tohidinezhad, Hugo C. Temperley, Mirac Ajredini, Bedirye Koyuncu Sokmen, Rumeysa Atabey, Leyla Ozer, Erman Aytac, Alison Corr, Alberto Traverso, James F. Meaney, Michael E. Kelly

**Affiliations:** ^1^ Department of Radiology St. James's Hospital Dublin Ireland; ^2^ School of Medicine Trinity College Dublin Dublin Ireland; ^3^ Centre for Advanced Medical Imaging (CAMI) St. James's Hospital Dublin Ireland; ^4^ Department of Radiation Oncology (Maastro Clinic), School for Oncology and Reproduction (GROW) Maastricht University Medical Centre Maastricht the Netherlands; ^5^ Acibadem University Atakent Hospital Gastrointestinal Oncology Unit Istanbul Turkey; ^6^ School of Medicine Libera Università Vita‐Salute San Raffaele Milan Italy; ^7^ Department of Surgery St. James's Hospital Dublin Ireland; ^8^ Trinity St. James Cancer Institute (TSJCI) St. James's Hospital Dublin Ireland

**Keywords:** advanced rectal cancer, MRI, oncology, Radiomics, recurrence

## Abstract

**Introduction:**

Local recurrence and distant metastasis remain a concern in advanced rectal cancer, with up to 10% and 20%–30% of patients suffering local and distal progression, respectively. Radiomics refers to a novel technology that extracts and analyses quantitative imaging features from images, which can be subsequently used to develop and test clinical models predictive of outcomes. We aim to develop and test an MRI‐based radiomics nomogram predictive of disease recurrence in patients with T4 rectal cancer.

**Methods:**

We conducted a multi‐institutional retrospective analysis of 55 patients with T4 rectal cancer treated with neoadjuvant chemoradiotherapy followed by exenterative surgery. Radiomic features were extracted from pre‐treatment T2‐weighted MRI scans and used to construct predictive models. The top‐performing radiomic signatures were identified, and internal validation with 1000 bootstrap samples was performed to calculate optimism‐corrected performance measures.

**Results:**

Two radiomic signatures were identified as strong predictors of post‐operative disease recurrence. The best‐performing model achieved an optimism‐corrected AUC of 0.75, demonstrating good discriminative ability. Calibration plots showed a satisfactory fit of the predictions to the actual rates, and decision curve analyses confirmed the positive net benefit of the models.

**Conclusion:**

The MRI‐based radiomics nomogram provides a promising tool for predicting disease recurrence in T4 rectal cancer patients post‐exenteration. This model could improve risk stratification and guide more personalized treatment strategies. Further studies with larger cohorts and external validation are needed to confirm these findings and enhance the model's generalizability.

## 1 Introduction

Neoadjuvant chemoradiotherapy (NACRT)/total neoadjuvant treatment followed by total mesorectal excision (TME) remains the cornerstone of treatment for locally advanced rectal cancer (LARC) [1]. TME refers to the meticulous dissection of the tumour, surrounding mesorectum and associated lymph nodes through the avascular embryologic plane, rapidly becoming the gold standard resection for LARC after its introduction in the 1980s [1, 2]. NACRT with 5‐fluorouracil (5‐FU) has been shown to improve organ preservation, decrease local recurrence and yield higher R0 rates in patients with LARC [3, 4, 5]. An alternative approach, total neoadjuvant therapy (TNT), refers to additional cycles of chemotherapy in the neoadjuvant period, resulting in more early intensified treatment [6]. TNT strategies lack consensus and vary substantially in radiation dose and fractions as well as timing of chemotherapeutic regimens [7]. Similarly to conventional NACRT, this aggressive approach promises improved systemic disease control, higher compliance and earlier stoma reversal, with the added benefit of potentially avoiding adjuvant therapy [7]. Despite these improvements, local recurrence and distant metastasis remain a concern, with up to 10% and 20%–30% of patients suffering local and distal progression respectively [8, 9]. Identifying prognostic indicators for disease recurrence may guide the therapeutic approach and enhance the patient counselling experience, such as offering a more aggressive treatment approach in patients deemed likely to recur based on pre‐therapeutic factors [10].

High‐resolution magnetic resonance imaging (MRI) remains the investigation of choice for evaluating tumour morphological characteristics and local staging in LARC [11]. Radiomics refers to a novel technology which extracts and analyses quantitative imaging features from images, which can be subsequently used to develop and test clinical models predictive of outcomes (e.g., survival, recurrence and response to treatment) [12, 13]. Previous studies have demonstrated that an MRI‐based radiomics signature (Rad) of the primary rectal tumour could be used to predict lymph node metastasis, tumour deposits and extranodal extension (ENE) with good performance [14, 15, 16, 17]. We hypothesise that pre‐operative prediction of recurrence (local or distal) from pre‐therapeutic imaging could be of great significance to treatment choice and enhance the patient counselling experience. Our study aims to develop and test a nomogram incorporating both clinicopathological and T2‐weighted MRI radiomics features predictive of disease recurrence in patients with LARC.

## 2 Materials and Methods

### 2.1 Participants

This multi‐institutional retrospective study was performed in accordance with the Declaration of Helsinki 1964 and approved by the SJH/TUH Joint Research Ethics Committee (JREC). Written informed consent was waived due to the retrospective design of the study.

From January 2013 to January 2021, a total of 55 consecutive matched patients with advanced rectal cancer from two centres (Institution 1: 44, Institution 2: 11) were enrolled in this study. Data from both institutions was pooled for radiomics analysis, and internal validation was performed using 1000 bootstrapping samples. Patients who met the following criteria were eligible for inclusion in our study.

#### 2.1.1 Inclusion

- Histologically confirmed rectal adenocarcinoma.
- Clinical staging: T4 +/− N+.
- Initial staging MRI of the rectum prior to any intervention available.
- Absence of metastatic disease (M0) at presentation.
- Treatment with curative intent.
- Resection margin status: R0, R1 or R2.

#### 2.1.2 Exclusion

- Palliative resection.
- Insufficient follow‐up data (3 years).
- Distant metastases (M1) at presentation.
- Recurrent rectal cancer.

### 2.2 Treatment and Follow‐Up

All included patients received neoadjuvant therapy and exenterative surgery as guided by a multidisciplinary discussion. The neoadjuvant treatment approaches varied due to differences in institutional protocols across the participating centres. Patients either received standard chemoradiotherapy (CRT) or TNT, with TNT regimens differing slightly in terms of sequencing and chemotherapy agents. Resected specimens were analysed according to local hospital protocol including TNM classification as per the American Joint Committee on Cancer staging system (8th edition). Post‐operative surveillance, consisting of digital rectal exam (DRE), endoscopy, CT‐TAP and rectal MRI, was performed at intervals dictated by local hospital protocol to evaluate for local and distal disease recurrence. Local recurrence was defined as a relapse occurring at the site of original surgical resection observed on endoscopy or MRI and proven histopathologically, whereas organ metastasis was confirmed by imaging and/or biopsy in all patients.

### 2.3 Imaging Acquisition and Segmentation

Routine staging rectal MRI was performed on patients using various scanners. Individual scanning parameters are outlined in the Table S1. MR imaging was collected using the PACS system. The selected sequence in our study was T2‐weighted imaging (T2WI). MRI images were retrospectively reviewed by Radiologist A (30 years in advanced pelvic cancers diagnostics) and confirmed by Radiologist B (15 years in advanced pelvic cancers diagnostics). Discrepancies between reviewers were resolved following discussion and joint consensus. Regions of interest (gross tumour volume) were delineated manually along the tumour border slice by slice to incorporate the entire rectal tumour and exclude intraluminal gas and fluid using 3D Slicer (v. 5.4.0).

### 2.4 Radiomics Feature Extraction

The radiomic feature extraction pipeline comprised the following steps: data conversion and pre‐processing, configuration of radiomic extraction, and feature extraction. Data conversion was handled by a custom Python script that transformed the original DICOM and RTSTRUCT images into the .nrrd format, which is compatible with pyradiomics. For this, the Python packages simpleITK v2.1 and pyplastimatch v1.9.3 were utilized to convert the DICOM CT images and the contours GTV, GTVp, GTVn into .nrrd images and corresponding binary masks. The configuration of radiomic extraction involved re‐sampling the original images to a uniform pixel spacing of [1] using B‐spline interpolation, eliminating outliers from the binary masks that exceeded 3σ from the intensity distribution for each patient, and applying wavelet filtering in all 13 directions to produce wavelet features. Features calculated for the original and wavelet‐filtered images included first‐order statistical features and texture matrices (GLCM, GLSZM, GLRLM, NGTDM). Morphological features were derived only from the original images. A fixed‐bin width approach (*n* = 25) was employed for quantising the statistical and texture features. Features were normalised to a *Z*‐score, a standard procedure in statistical analyses.

### 2.5 Clinical and Histopathological Variables

Other potential clinical factors for post‐operative disease recurrence were recorded and investigated, including age, sex, cN stage, tumor differentiation grade and distance from the anal verge.

### 2.6 Model Development

The process for selecting radiomic features was adapted from previous studies conducted by members of our research group [18, 19]. Data from both institutions was pooled for radiomics analysis, and internal validation was performed using 1000 bootstrapping samples. In summary, the following four steps were followed to identify the most predictive radiomic features. Firstly, 1000 bootstrap samples with replacement were drawn from the original cohort. In each bootstrap sample, pairwise mean absolute correlations were calculated to reduce the number of highly correlated features (*r* > 0.9 or *r* < −0.9) in an unsupervised manner. Secondly, the least absolute shrinkage and selection operator (LASSO) embedded with logistic regression using 5‐fold cross‐validation was applied to the 1000 samples to rank the features according to their retention frequency by LASSO. Next, the top six features were arbitrarily selected based on the decrease in frequency of the selected features. It is important to note that among the same features with different wavelet decompositions, the one with the highest frequency was chosen. Additionally, the selected top radiomic features were compared across different numbers of bootstrap samples. The six selected features were then used to perform stepwise backward logistic regression on the same 1000 bootstrap samples. Finally, the most frequently occurring signature (comprising more than one radiomic feature) was arbitrarily selected to construct the final model. The original cohort was utilized to fit the coefficients of the final model.

The clinical model was trained using the standard stepwise backward logistic regression on the five candidate predictors. Significant predictors in both the clinical and radiomic models, with the highest effect estimates based on odds ratio (OR), were chosen to build the combined model. To avoid overfitting, a maximum of three predictors was included in a single model (event per variable > 10) [20].

### 2.7 Model Evaluation

The discrimination power of the prediction models was assessed using the area under the receiver operating characteristic curve (AUC). Sensitivity, specificity, positive predictive value, negative predictive value and accuracy were also calculated based on the threshold determined by the Youden index method. Calibration, which measures the agreement between predicted probabilities and actual outcomes, was evaluated using a graphical assessment of calibration (a scatter plot where the *x* = *y* line indicates perfect calibration) [21]. Internal validation with 1000 bootstrap samples was conducted to estimate the statistical optimism of the AUCs and calibration slopes, following the method recommended in the Transport Reporting of a Multivariable Prediction Model for Individual Prognosis or Diagnosis guidelines [22, 23]. The estimated optimisms were subtracted from the original AUCs and calibrations to obtain the optimism‐corrected performance measures.

Decision curve analysis was employed to visualise the decisional benefit of the models, considering ‘Treat All’ and ‘Treat None’ as the benchmark strategies. This method calculates the net benefit as a function of the relative harms associated with false predictions across a range of threshold probabilities. The model with the highest net benefit at a particular threshold is considered optimal, regardless of the magnitude of the difference [24].

To facilitate the use of the prediction model in future diagnostic research, a nomogram was developed to predict the probability of having post‐operative disease recurrence using radiomic features. All analyses were performed using R v.4.2.2 (R Foundation for Statistical Computing).

## 3 Results

### 3.1 Patient Characteristics

Patient characteristics are presented in Table 1. Median follow‐up time was 35 (10–160) and 67 (15–94) months in centres 1 and 2, respectively. Eighteen (41%) patients suffered disease recurrence in centre 1, compared with five (45%) patients in centre 2.

**TABLE 1:** Descriptive statistics of the study sample.

Characteristic	Total (*N* = 55)	Centre 1 (*n* = 44)	Centre 2 (*n* = 11)	*p*
Age				
< 60	21 (38%)	15 (34%)	6 (55%)	0.3
≥ 60	34 (62%)	29 (66%)	5 (45%)	
Male gender	33 (60%)	27 (61%)	6 (55%)	0.7
cN stage				
0	18 (33%)	17 (39%)	1 (9.1%)	*0.05
1	19 (35%)	12 (27%)	7 (64%)	
2–3	18 (33%)	15 (34%)	3 (27%)	
Differentiation grade				
Moderate	44 (80%)	34 (77%)	10 (91%)	0.6
Poor	6 (11%)	6 (14%)	0 (0%)	
Well	5 (9.1%)	4 (9.1%)	1 (9.1%)	
Distance from anal verge (cm)				
< 9	32 (58%)	22 (50%)	10 (91%)	*0.02
≥ 9	23 (42%)	22 (50%)	1 (9.1%)	

*Note:* Values presented as *n* (%). Comparisons were performed using Fisher's exact test or Pearson's chi‐squared test.

Abbreviation: cN, clinical nodal status.

### 3.2 Prediction Models

Following analysis, none of the clinical factors were found to be independent predictors of post‐operative disease recurrence. The two top‐performing signatures based on T2‐MRI for predicting the risk of post‐operative disease recurrence in rectal cancer are demonstrated in Table 2.

**TABLE 2:** Best performing radiomic signatures based on T2‐weighted magnetic resonance imaging (T2‐MRI) for predicting the risk of recurrence in patients with rectal cancer treated with neoadjuvant chemoradiotherapy and surgical resection (*n* = 55).

	OR (95% CI)	*p*
Signature 1		
(Intercept)	0.66 (0.36–1.22)	0.2
Wavelet HHL glcm cluster shade	0.34 (0.14–0.83)	*0.018
Wavelet LLL glszm small area high grey level emphasis	0.43 (0.22–0.85)	*0.016
Wavelet HHH firstorder skewness	0.41 (0.17–0.96)	*0.040
Signature 2		
(Intercept)	0.66 (0.35–1.26)	0.2
Wavelet LLL glcm difference variance	0.25 (0.10–0.66)	*0.005
Wavelet LHH gldm small dependence low grey level emphasis	2.28 (0.93–5.56)	0.071
Original shape surface volume ratio	2.49 (0.95–6.50)	0.063

*denotes statistically significant p‐value.

### 3.3 Model Evaluation

**FIGURE 1:**
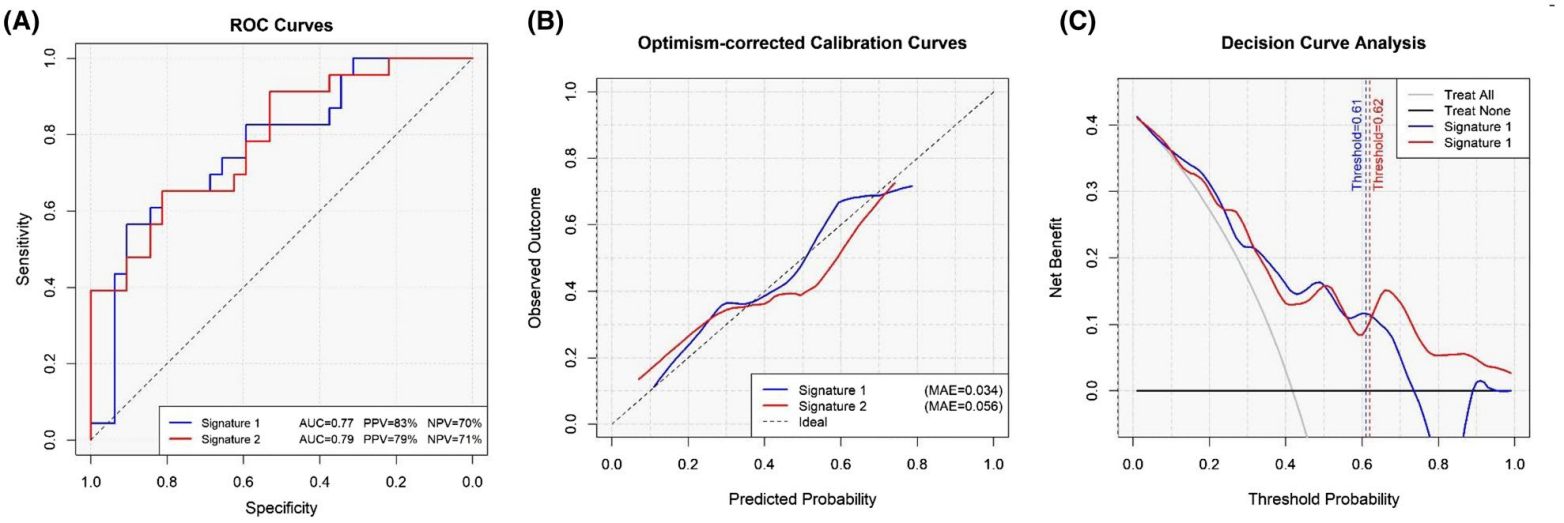
(A–C) Discrimination, calibration and decision curve analysis of the radiomic signatures using internal validation with 1000 bootstrap resamples (Optimism‐corrected AUCs: signature 1: 0.73, signature 2: 0.75).

**FIGURE 2:**
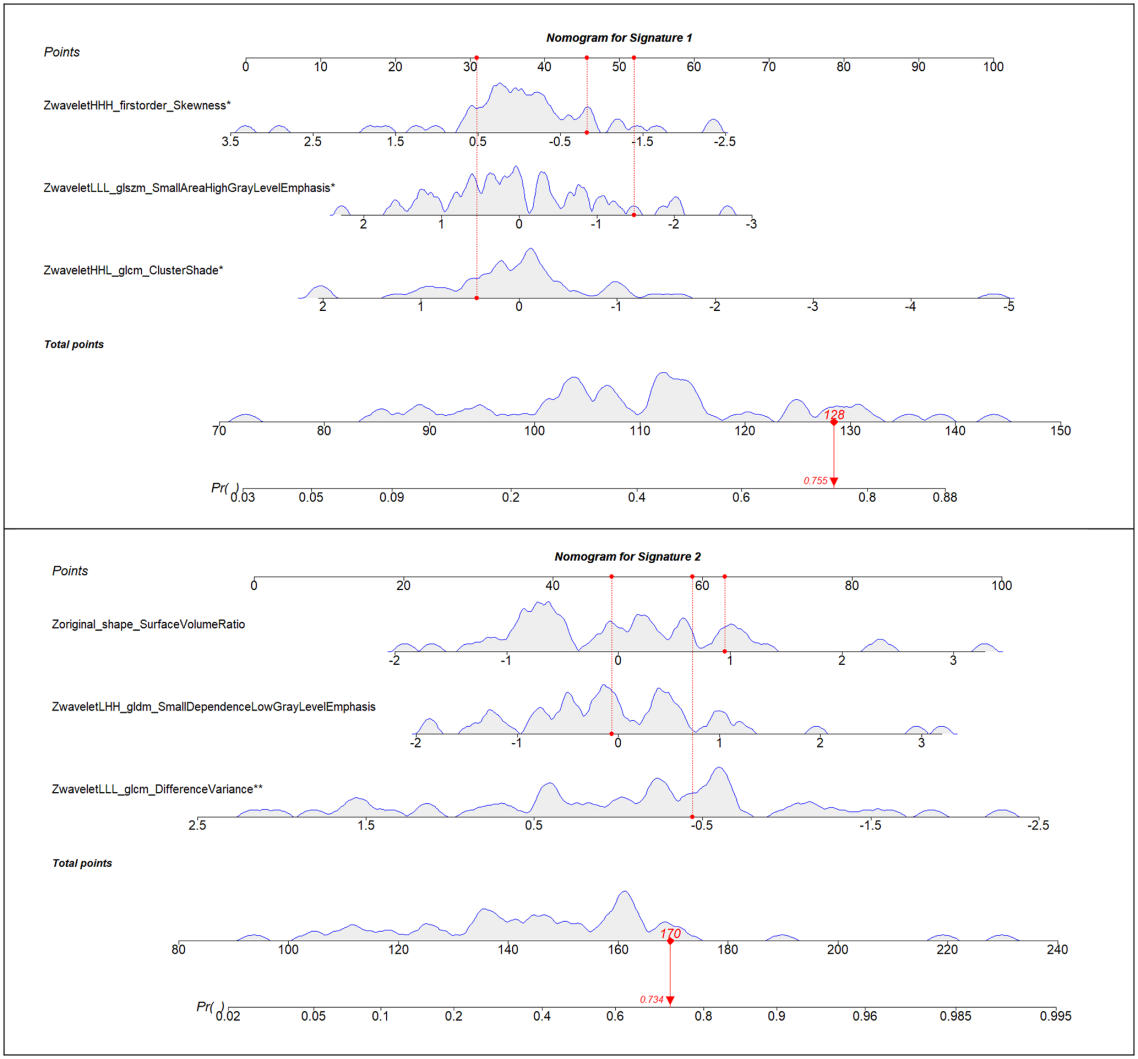
Nomograms of the radiomics signatures reported in Table 2.

As shown in Figure 1A, the radiomic signature 2 with an optimism‐corrected AUC of 0.75 had the highest performance. The negative predictive value and positive predictive value of this signature were 71% and 79%, respectively. The calibration power of the two models is shown in Figure 1B. The optimism‐corrected calibration curves for both signatures indicate that the models provide well‐calibrated predictions of post‐operative disease recurrence in patients with advanced rectal cancer, supporting the robustness and clinical applicability of these signatures. Additionally, the decision curve analysis (Figure 1C) revealed the higher net benefit of signature 2 across the entire range of threshold probabilities when compared to signature 1. To facilitate the application of the prediction models in future diagnostic research, the nomograms of signatures 1 and 2, presenting the probability estimation for a sample patient, are demonstrated in Figure 2A,B. To improve the reporting standards of the paper, all data have been presented according to the Transport Reporting of a Multivariable Prediction Model for Individual Prognosis or Diagnosis (TRIPOD) guidelines (Table S2; [25]).

## 4 Discussion

To the best of our knowledge, this study represents the first patient‐specific risk prediction algorithm to estimate the probability of post‐operative disease recurrence in patients undergoing NACRT and surgical resection for T4 rectal cancer. While a lack of independent clinical predictors prevented the development of a clinical model, our analysis produced two radiomics‐based signatures with significant predictive value of recurrence in this cohort of patients. In our study, a total of six features derived from T2WI MRI with high stability, low redundancy, and a close relationship with post‐operative disease recurrence in this cohort of patients were utilised to develop the two independent radiomics signatures. Each model based on identified features showed good performance in the prediction of recurrence using internal validation with 1000 bootstrapping samples, corresponding to an optimism‐corrected AUC of 0.73 and 0.75 in signatures 1 and 2, respectively.

MRI‐based radiomics signatures have consistently demonstrated good performance in rectal cancer research, particularly in the prediction of treatment response, lymph node status, and disease recurrence [14, 26, 27, 28, 29, 30, 31, 32]. Chen et al. [31] investigated the significance of MRI‐based radiomics in differentiating between local recurrence and nonrecurrence lesions at the site of anastomosis. A combined model consisting of T2WI, diffusion‐weighted imaging (DWI) and T1‐weighted volume interpolated body examination (VIBE) sequences demonstrated excellent performance, with reported AUC, sensitivity and specificity of 0.864, 81.82% and 75.86%, respectively. Interestingly, the combined model performed better than individual sequence models, suggesting a benefit to performing radiomics analysis on regions of interest from multiple different sequences. Recently, Li et al. published the results of their study investigating the utility of T2WI‐based MRI radiomics in the prediction of pre‐operative ENE and prognosis in patients with resectable rectal cancer [14]. The constructed nomogram, consisting of RadScore, age, cT stage, lymph node border irregularity and adjacent fat invasion, achieved an AUC of 0.799 and 0.736 in the training and validation models, respectively. Nomogram‐based ENE was subsequently demonstrated to be an independent risk factor for 3‐year recurrence‐free survival.

Advanced rectal cancer poses significant challenges in terms of management and prognosis [33]. Despite advancements in treatment modalities such as NACRT/total neoadjuvant treatment followed by surgical resection, the outcomes for patients with advanced rectal cancer remain variable [34]. The need for robust prognostic indicators to guide treatment decisions and monitor response in this cohort of patients is paramount [35]. Although several clinicopathological factors (MRI and endoscopic response) are currently used to stratify response to treatments in patients with advanced rectal cancer, they have limitations [36]. In addition, traditional prognostic factors such as tumour stage and histological grade can provide circumstantial value about potential disease behaviour and treatment response. There is a need to identify novel biomarkers that can complement existing prognostic tools and enhance risk stratification in LARC [37]. By extracting and analyzing quantitative features from medical images, radiomics offers the potential to uncover hidden patterns and relationships that can serve as predictive markers and ultimately facilitate a more personalised treatment approach and surveillance strategy [38].

The following limitations should be taken into account when interpreting the results from this study. Given the relatively infrequent treatment of LARC over an eight‐year period within the two included institutions, our overall sample size was small. Due to the retrospective and multi‐centre nature of our study, certain clinical data such as lymphovascular invasion, perineural invasion, BMI and mean nodal yield were inconsistently reported across institutions. For this reason, we did not include these variables in our analysis to avoid introducing bias or drawing unreliable conclusions. Secondly, the few numbers of events per centre resulted in the inability to perform external validation. Despite this, bootstrapping‐adjusted performance measures were utilised to correct the optimistic results of internal validation. Similarly, we were unable to develop and test the performance of a combined nomogram, as no independent clinical predictors of post‐operative disease recurrence could be identified.

The major practical implications of our study are that it presents an empirical, data‐driven prediction model which has provided acceptable patient‐level predictions for identifying post‐operative disease recurrence events. We believe that the identification of patients deemed to be at higher risk of post‐operative disease recurrence will enhance the patient counselling experience, as well as justify a more radical resection, adjuvant therapy or more intensive surveillance in the post‐operative period. The current research is timely, given the increasing ‘radicalness’ of exenterative surgery, continually pushing the boundaries of what was previously deemed unresectable. Moreover, due to the multi‐institutional design of this study, our results are likely to be pertinent to similar centres. Despite this, further studies with large cohorts and external validation are required to further validate our findings. The authors plan to expand our analysis of radiomics in advanced rectal cancer via collaboration with other centres to assemble a larger, independent cohort for validation of our radiomics model. Additionally, we aim to incorporate datasets from diverse populations and imaging platforms to enhance the model's applicability across different clinical settings.

## 5 Conclusion

The T2WI‐based radiomics signatures developed in our study demonstrated good performance in predicting post‐operative disease recurrence in patients with advanced rectal cancer. Further large cohort multi‐institutional studies are required to corroborate our findings.

## Author Contributions

**Niall J. O'Sullivan:** conceptualization (equal), data curation (equal), formal analysis (equal), investigation (equal), methodology (equal), validation (equal), writing – original draft (equal), writing – review and editing (equal). **Fariba Tohidinezhad:** conceptualization (equal), data curation (equal), formal analysis (equal), investigation (equal), methodology (equal), resources (equal), software (equal), writing – original draft (equal). **Hugo C. Temperley:** conceptualization (equal), data curation (equal), investigation (equal), writing – original draft (equal). **Mirac Ajredini:** conceptualization (equal), data curation (equal), formal analysis (equal), writing – original draft (equal). **Bedirye Koyuncu Sokmen:** conceptualization (equal), data curation (equal), formal analysis (equal), writing – original draft (equal). **Rumeysa Atabey:** conceptualization (equal), data curation (equal), writing – original draft (equal). **Leyla Ozer:** conceptualization (equal), data curation (equal), writing – original draft (equal). **Erman Aytac:** conceptualization (equal), data curation (equal), writing – original draft (equal), writing – review and editing (equal). **Alison Corr:** conceptualization (equal), formal analysis (equal), resources (equal), supervision (equal), writing – original draft (equal), writing – review and editing (equal). **Alberto Traverso:** conceptualization (equal), data curation (equal), formal analysis (equal), software (equal), writing – original draft (equal), writing – review and editing (equal). **James F. Meaney:** conceptualization (equal), data curation (equal), formal analysis (equal), writing – original draft (equal), writing – review and editing (equal). **Michael E. Kelly:** conceptualization (equal), data curation (equal), supervision (equal), writing – original draft (equal), writing – review and editing (equal).

## Conflicts of Interest

The authors declare no conflicts of interest.

## Supporting information


Data S1.

## Data Availability

The authors confirm that the data supporting the findings of this study are available within the article and its Supporting Information.
