# Supplementary material for: Multi‐Institutional MR‐Derived Radiomics to Predict Post‐Exenteration Disease Recurrence in Patients With T4 Rectal Cancer
Source: Cancer Med. 2025 Feb 18;14(4):e70699. doi: 10.1002/cam4.70699 (PMC11836347; doi:10.1002/cam4.70699)
Supplement: Supplementary file 1 — Data S1. [file CAM4-14-e70699-s001.docx]

**Supporting Information**

**Table S1: Image acquisition protocols**

Institution 1

| **Patient number** | **MRI Model** | **Field Strength (Tesla)** | **Field of View** | **Echo Time** | **Repetition Time** | **Slice thickness** | **Slice interval** | **Zone Matrix** | **Contrast agent (if applicable)** | **Contrast dose (if applicable)** | **Rate of contrast injection (if applicable)** |
| --- | --- | --- | --- | --- | --- | --- | --- | --- | --- | --- | --- |
| 1 | GE Signa Excite | 1.5 | - | 91 | 5900 | 4mm | 4.4 | 320x224 |  |  |  |
| 2 | Philips Achieva | 1.5 | - | 100 | 2873 | 3mm | 4mm | 280x220 |  |  |  |
| 3 | Philips Achieva | 1.5 | - | 100 | 3365 | 3mm | 3.3mm | 280x219 |  |  |  |
| 4 | Philips Achieva | 1.5 | - | 100 | 3141 | 3mm | 3.4mm | 280x219 |  |  |  |
| 5 | Philips Achieva | 1.5 | - | 100 | 2873 | 3mm | 3.3mm | 280x220 |  |  |  |
| 6 | Siemens Magnetom | 1.5 | - | 82 | 6230 | 3mm | 3.9mm | 256x224 |  |  |  |
| 7 | Siemens Symphony | 1.5 | - | 94 | 5150 | 3mm | 3.9mm | 256x179 |  |  |  |
| 8 | Siemens Magnetom | 3 | - | 104 | 7990 | 5mm | 5mm | 368x313 |  |  |  |
| 9 | Philips Achieva | 1.5 | - | 100 | 2173 | 3mm | 3.4mm | 308x226 |  |  |  |
| 10 | Siemens Symphony | 1.5 | - | 94 | 4760 | 3mm | 3.9mm | 256x179 |  |  |  |
| 11 | Philips Achieva | 1.5 | - | 100 | 2873 | 3mm | 3.3mm | 280x220 |  |  |  |
| 12 | Philips Achieva | 3 | - | 110 | 4971 | 3mm | 3.3mm | 232x193 |  |  |  |
| 13 | Philips Achieva | 1.5 | - | 100 | 4227 | 4mm | 5mm | 384x297 |  |  |  |
| 14 | Philips Achieva | 1.5 | - | 100 | 2340 | 3mm | 3.3mm | 288x224 |  |  |  |
| 15 | Philips Achieva | 1.5 | - | 120 | 2922 | 4mm | 4.4mm | 348x256 |  |  |  |
| 16 | Philips Achieva | 1.5 | - | 100 | 2916 | 3mm | 3.6mm | 280x220 |  |  |  |
| 18 | Siemens Symphony | 1.5 | - | 94 | 5150 | 3mm | 3.9mm | 256x179 |  |  |  |
| 19 | Philips Achieva | 1.5 | - | 100 | 2916 | 3mm | 3.3mm | 280x219 |  |  |  |
| 20 | Philips Achieva | 1.5 | - | 100 | 2916 | 3mm | 3.3mm | 280x219 |  |  |  |
| 21 | Philips Achieva | 1.5 | - | 90 | 4398 | 3mm | 3.5mm | 260x196 |  |  |  |
| 22 | Siemens Aera | 1.5 | - | 100 | 7570 | 4mm | 4mm | 256x220 |  |  |  |
| 23 | Siemens magnetom | 3 | - | 104 | 7500 | 5mm | 5mm | 368x313 |  |  |  |
| 24 | Philips Achieva | 1.5 | - | 100 | 3094 | 3mm | 3.3mm | 280x220 |  |  |  |
| 25 | Philips Achieva | 1.5 | - | 100 | 3365 | 3mm | 3.6mm | 232x176 |  |  |  |
| 26 | Philips Achieva | 1.5 | - | 100 | 2287 | 3mm | 3.5mm | 280x219 |  |  |  |
| 27 | Philips Achieva | 1.5 | - | 100 | 2916 | 3mm | 3.3mm | 280x219 |  |  |  |
| 28 | Philips Achieva | 1.5 | - | 100 | 4038 | 3mm | 3.3mm | 232x176 |  |  |  |
| 29 | GE discovery | 3 | - | 153 | 5741 | 4.5mm | 4.9mm | 288x224 |  |  |  |
| 30 | Philips Achieva | 1.5 | - | 100 | 3141 | 3mm | 3.3mm | 280x219 |  |  |  |
| 31 | Siemens Symphony | 1.5 | - | 94 | 4760 | 3mm | 3.6mm | 256x179 |  |  |  |
| 32 | Philips Achieva | 1.5 | - | 100 | 3365 | 3mm | 3.8mm | 280x220 |  |  |  |
| 33 | Philips Achieva | 1.5 | - | 100 | 3094 | 3mm | 3.3mm | 280x220 |  |  |  |
| 34 | Philips Achieva | 1.5 | - | 90 | 4398 | 3mm | 3.5mm | 260x196 |  |  |  |
| 35 | Philips Achieva | 1.5 | - | 90 | 4215 | 4mm | 4.4mm | 348x341 |  |  |  |
| 36 | Philips Achieva | 1.5 | - | 100 | 2873 | 3mm | 3.3mm | 280x220 |  |  |  |
| 37 | Philips Achieva | 1.5 | - | 90 | 4001 | 4mm | 4.4mm | 224x224 |  |  |  |
| 38 | Philips Achieva | 1.5 | - | 90 | 3106 | 3mm | 3.3mm | 224x224 |  |  |  |
| 39 | Philips Achieva | 1.5 | - | 100 | 3315 | 3mm | 3.3mm | 232x176 |  |  |  |
| 40 | Siemens Aera | 1.5 | - | 100 | 8930 | 4mm | 4mm | 256x227 |  |  |  |
| 41 | Philips Achieva | 1.5 | - | 100 | 3285 | 3mm | 3.6mm | 320x217 |  |  |  |
| 42 | Philips Achieva | 1.5 | - | 100 | 2916 | 3mm | 3.3mm | 280x219 |  |  |  |
| 43 | Philips Achieva | 1.5 | - | 100 | 2692 | 3mm | 3.3mm | 280x220 |  |  |  |
| 44 | GE signa HDxt | 1.5 | - | 104 | 4680 | 4mm | 5mm | 384x224 |  |  |  |

Institution 2

| **Patient number** | **MRI Model** | **Field Strength (Tesla)** | **Field of View** | **Echo Time** | **Repetition Time** | **Slice thickness** | **Slice interval** | **Zone Matrix** | **Contrast agent (if applicable)** | **Contrast dose (if applicable)** | **Rate of contrast injection (if applicable)** |
| --- | --- | --- | --- | --- | --- | --- | --- | --- | --- | --- | --- |
| 1 | siemens symphonyTim | 1.5T | 200 | 101 | 4320 | 3.5 | 30 | 320x320 | Dotarem | 12cc | 2 ml per second |
| 2 | siemens symphonyTim | 1.5T | 200 | 101 | 4320 | 3.5 | 30 | 320x320 | Gadovist | 12cc | 2 ml per second |
| 3 | siemens symphonyTim | 1.5T | 200 | 101 | 4320 | 3.5 | 30 | 320x320 | Gadovist | 12cc | 2 ml per second |
| 4 | siemens symphonyTim | 1.5T | 200 | 101 | 4320 | 3.5 | 30 | 320x320 | Gadovist | 12cc | 2 ml per second |
| 5 | siemens symphonyTim | 1.5T | 200 | 101 | 4320 | 3.5 | 30 | 320x320 | Gadovist | 12cc | 2 ml per second |
| 6 | siemens symphonyTim | 1.5T | 200 | 101 | 4320 | 3.5 | 30 | 320x320 | Gadovist | 12cc | 2 ml per second |
| 7 | siemens symphonyTim | 1.5T | 200 | 101 | 4320 | 3.5 | 30 | 320x320 | Gadovist | 12cc | 2 ml per second |
| 8 | siemens symphonyTim | 1.5T | 200 | 101 | 4320 | 3.5 | 30 | 320x320 | Gadovist | 12cc | 2 ml per second |
| 9 | siemens symphonyTim | 1.5T | 200 | 101 | 4320 | 3.5 | 30 | 320x320 | Gadovist | 12cc | 2 ml per second |
| 10 | siemens symphonyTim | 1.5T | 200 | 101 | 4320 | 3.5 | 30 | 320x320 | Gadovist | 12cc | 2 ml per second |
| 11 | siemens symphonyTim | 1.5T | 200 | 101 | 4320 | 3.5 | 30 | 320x320 | Gadovist | 12cc | 2 ml per second |

| **Section/Topic** | **Item** |  | **Checklist Item** | **Page** |
| --- | --- | --- | --- | --- |
| **Title and abstract** | | | | |
| Title | 1 | D;V | Identify the study as developing and/or validating a multivariable prediction model, the target population and the outcome to be predicted. | 1 |
| Abstract | 2 | D;V | Provide a summary of objectives, study design, setting, participants, sample size, predictors, outcome, statistical analysis, results and conclusions. | 2 |
| **Introduction** | | | | |
| Background and objectives | 3a | D;V | Explain the medical context (including whether diagnostic or prognostic) and rationale for developing or validating the multivariable prediction model, including references to existing models. | 3 |
|  | 3b | D;V | Specify the objectives, including whether the study describes the development or validation of the model or both. | 3 |
| **Methods** | | | | |
| Source of data | 4a | D;V | Describe the study design or source of data (e.g., randomized trial, cohort, or registry data), separately for the development and validation data sets, if applicable. | 4 |
|  | 4b | D;V | Specify the key study dates, including start of accrual; end of accrual; and, if applicable, end of follow-up. | 4 |
| Participants | 5a | D;V | Specify key elements of the study setting (e.g., primary care, secondary care, general population) including number and location of centers. | 4 |
|  | 5b | D;V | Describe eligibility criteria for participants. | 4 |
|  | 5c | D;V | Give details of treatments received, if relevant. | 4/5 |
| Outcome | 6a | D;V | Clearly define the outcome that is predicted by the prediction model, including how and when assessed. | 5 |
|  | 6b | D;V | Report any actions to blind assessment of the outcome to be predicted. | 5 |
| Predictors | 7a | D;V | Clearly define all predictors used in developing or validating the multivariable prediction model, including how and when they were measured. | 5 |
|  | 7b | D;V | Report any actions to blind assessment of predictors for the outcome and other predictors. | 6 |
| Sample size | 8 | D;V | Explain how the study size was arrived at. | 4 |
| Missing data | 9 | D;V | Describe how missing data were handled (e.g., complete-case analysis, single imputation, multiple imputation) with details of any imputation method. | 5/6 |
| Statistical analysis methods | 10a | D | Describe how predictors were handled in the analyses. | 6/7 |
|  | 10b | D | Specify type of model, all model-building procedures (including any predictor selection) and method for internal validation. | 6/7 |
|  | 10c | V | For validation, describe how the predictions were calculated. | NA |
|  | 10d | D;V | Specify all measures used to assess model performance and, if relevant, to compare multiple models. | 7 |
|  | 10e | V | Describe any model updating (e.g., recalibration) arising from the validation, if done. | NA |
| Risk groups | 11 | D;V | Provide details on how risk groups were created, if done. | NA |
| Development vs. validation | 12 | V | For validation, identify any differences from the development data in setting, eligibility criteria, outcome and predictors. | NA |
| **Results** | | | | |
| Participants | 13a | D;V | Describe the flow of participants through the study, including the number of participants with and without the outcome and, if applicable, a summary of the follow-up time. A diagram may be helpful. | Table 1 |
|  | 13b | D;V | Describe the characteristics of the participants (basic demographics, clinical features, available predictors), including the number of participants with missing data for predictors and outcome. | Table 1 |
|  | 13c | V | For validation, show a comparison with the development data of the distribution of important variables (demographics, predictors and outcome). | NA |
| Model development | 14a | D | Specify the number of participants and outcome events in each analysis. | 8 |
|  | 14b | D | If done, report the unadjusted association between each candidate predictor and outcome. | NA |
| Model specification | 15a | D | Present the full prediction model to allow predictions for individuals (i.e., all regression coefficients and model intercept or baseline survival at a given time point). | 10 |
|  | 15b | D | Explain how to the use the prediction model. | 9 |
| Model performance | 16 | D;V | Report performance measures (with CIs) for the prediction model. | 9 |
| Model-updating | 17 | V | If done, report the results from any model updating (i.e., model specification, model performance). | NA |
| **Discussion** | | | | |
| Limitations | 18 | D;V | Discuss any limitations of the study (such as nonrepresentative sample, few events per predictor, missing data). | 13 |
| Interpretation | 19a | V | For validation, discuss the results with reference to performance in the development data and any other validation data. | NA |
|  | 19b | D;V | Give an overall interpretation of the results, considering objectives, limitations, results from similar studies and other relevant evidence. | 13 |
| Implications | 20 | D;V | Discuss the potential clinical use of the model and implications for future research. | 13 |
|  | | | | |
| Supplementary information | 21 | D;V | Provide information about the availability of supplementary resources, such as study protocol, Web calculator and data sets. | NA |
| Funding | 22 | D;V | Give the source of funding and the role of the funders for the present study. | 1 |

**Table S2:** TRIPOD checklist for prediction model development and validation
